# Supplementary material for: Anthrax, livelihood vulnerability, and food insecurity in selected game management areas in Zambia: A mixed-methods analysis at the human-wildlife-livestock interface
Source: PLoS Negl Trop Dis. 2025 Jun 27;19(6):e0012893. doi: 10.1371/journal.pntd.0012893 (PMC12221164; doi:10.1371/journal.pntd.0012893)
Supplement: Attachment — Submitted filename: Reviewer Comments 00145.docx [file pntd.0012893.s002.docx]

**Responses to Reviewers’ Comments**

**Manuscript Title:** *Anthrax, Livelihood Vulnerability, and Food Insecurity in Selected Game Management Areas in Zambia: A Mixed-Methods Analysis at the Human-Wildlife-Livestock Interface*

We sincerely thank the reviewers for their detailed and constructive comments, which have significantly improved the quality of our manuscript. Below, we provide point-by-point responses. Changes made in the manuscript are indicated by line numbers.

**Reviewer #2:** The objectives and hypothesis are not clear, albeit indicated. Based on this, the study design is appropriate, but all descriptions need improvement. Statistics are used for demographic data, unnecessarily, but not presented for other data. There is no information about ethical or regulatory requirements.

Response: thank you for this comment. We have improved the descriptions. We have since removed the unnecessary statistics and kept the study descriptive and exploratory. We have included the ethical approve statement. All research procedures complied with applicable ethical and regulatory guidelines for human subjects research in Zambia.

**Detailed comments:**

**Comment (L116–120):** These sentences are wordy and difficult to follow. It would help if you could specify which aspects are associated with animal outbreaks and which are connected to human outbreaks, and which can be linked to both. In addition, it is unclear how you examine how human-wildlife interactions have effects on livestock management, what is the hypothesis behind this statement?

Response: Thank you for this helpful observation. The underlying hypothesis is that close proximity to wildlife and shared use of environmental resources—such as grazing lands, watering points, and wildlife corridors—increase contact between domestic animals and wildlife. This proximity may lead to changes in livestock behavior (e.g., shifting grazing routes), elevate the risk of disease transmission (e.g., through contaminated carcasses or soil), and influence farmer decision-making (e.g., delaying reporting or selling sick animals). We examined this by integrating focus group discussions on daily livestock practices with survey data on herd movements, disease awareness, and resource use, thereby identifying how wildlife presence affects livestock management choices and anthrax exposure risk.

**Comment:** It is somewhat contradictory that the topic is presented as sensitive (L128), and therefore anonymization is needed.

**Response:** We appreciate this important observation. In the Zambian context, the illegal game meat trade is highly sensitive due to existing punitive legal frameworks. This sensitivity discourages poachers from openly participating in interviews for fear of prosecution. Therefore, we ensured the anonymity of responses to protect participants. We have rephrased the paragraph for greater clarity and coherence. Otherwise, we have revised the sentence and deleted the sensitivity statement.

**Comment (L132):** But study participants were still expected to provide their collective insights and diverse perspectives in a truthful way (L143). Given this, some justification for choosing the FGD methodology is needed.

**Response:** Thank you for this comment. We have revised the relevant sentences to better justify the use of focus group discussions. Given the sensitivity of the subject and the need for collective perspectives, FGDs were appropriate for facilitating open and context-rich dialogue. “We employed a mixed-method research design, combining qualitative and quantitative data collection approaches. Focus Group Discussions (FGDs) were selected as a critical qualitative method because they allow for the exploration of shared experiences, cultural norms, and community-level insights that may not surface through individual interviews or surveys. In rural settings where decision-making and coping strategies are often communal, FGDs are particularly well-suited to capturing group-level dynamics, consensus, and dissent on issues such as anthrax risk, food insecurity, and livelihood adaptation. This method encouraged open dialogue while preserving anonymity through group-based storytelling, enabling participants to reflect on complex, sensitive topics—including illegal meat trade, wildlife contact, and carcass handling—in a contextually safe and non-threatening environment. Although individual narratives may be diluted in a group setting, the collective perspectives offered rich, context-specific insights essential for understanding local disease dynamics and resilience strategies”.

**Comment (L161–162):** It is not only the human-wildlife-livestock interaction that makes anthrax thrive. I suggest toning down this aspect somewhat and rephrasing.

**Response:** Thank you for this suggestion. We agree and have revised the statement to reflect that the human-wildlife-livestock interface is one of several contributing factors to anthrax outbreaks. Please see the updated text in the revised manuscript.

**Comment (L172):** The sentence about understanding disease dynamics at the interface is a better argument for choosing the study areas. The choice of these particular national parks is still not very obvious.

**Response:** Thank you for pointing this out. We have provided additional justification for selecting the study areas. Lochinvar and Blue Lagoon National Parks were chosen due to recent anthrax outbreaks originating from wildlife, particularly hippos, in the Southern Province. Simalaha Community Conservancy was selected because anthrax is endemic in the Western Province. These areas also host the largest livestock populations in Zambia, making them critical interfaces for understanding disease dynamics.

Figure 1 is a little difficult to interpret without close inspection, some better highlighting of the study area is needed. Figure 2 is also difficult to interpret for a reader not familiar with a map of Zambia. I suggest making one map of Zambia with all study sites clearly marked while also highlighting the areas of interest that are indicated in the current maps.

Response: thank you for the comments. We have revised the figures 1 and 2 and put them in one map. The figures are now very clear.

L179: the aspects highlighted on this line are very important for anthrax but not necessarily for other zoonoses, so the sentence may need some rephrasing. I would prefer if the specific risk factors for anthrax was clarified in one place and the general risks for zoonotic transmission described as such. Now there is a mix of transmission aspects that are relevant for many zoonotic diseases, and environmental aspects that are particularly relevant for anthrax but described as if applicable for various zoonoses.

**Response:** We appreciate this feedback. The sentence has been revised accordingly.

L183-185: here it is already presumed that the interaction in itself amplifies the risk of outbreaks, while it was stated that the drivers of outbreaks would be investigated in the study. Do you mean that the disease is transmitted directly between these populations and, if yes, based on what evidence?

Response: Thank you for the question. We have include “may”. This proximity to wildlife may elevate the risk of anthrax outbreaks by increasing opportunities for indirect transmission between wildlife and livestock, such as through contaminated grazing areas, shared water sources, or contact with infected carcasses. While the precise mechanisms of transmission remain complex, this study investigates whether patterns of human-wildlife-livestock interaction in GMAs are associated with increased exposure risks and altered livestock management practices. The aim is to explore how ecological, behavioral, and socio-economic factors intersect to influence outbreak dynamics.

L204-207: this sentence needs rephrasing. How can determining saturation of the data involve recruiting FGD participants and conducting FGDs? I presume that data saturation was determined by continuous examination of the data as it emerged from each FGD but that is not what is stated here.

**Response:** Thank you for the clarification. We have rephrased the sentence to reflect that data saturation was monitored through ongoing review during FGDs, not through the recruitment process itself.

Table 1 and 2 could be put in supplementary material, as they do not contribute substantially to the understanding of the study.

Response: Thank you for the comment. These have been merged and moved to supplementary material.

L246-247 and L250: the logic of gatekeepers recruiting active members of the community and them sharing the agenda with the school teachers is hard to follow. Wsa this because the FGDs took place on the school premises, or because school teachers were involved in the recruitment? Pleas clarify the role of the headteachers and the precise method of participant recruitment by the gatekeepers.

**Response:** Thank you for this observation. We have deleted the sentence on the teachers as they had no direct role in the study other than provide a classroom for focus group discussions.

**Comment (L253–254):** Please delete the sentence "We then conducted a focus group meeting."

**Response:** Thank you for this comment. The sentence has been removed from the manuscript.

**Comment (Section 2.5.1):** Why was there no multivariable analysis? Please justify.

**Response:** Thank you for this valuable suggestion. Given the exploratory nature of our study, we focused on descriptive and bivariate analyses (Chi-square tests) to identify associations. We agree that multivariable analyses could offer additional insights and suggest including them in future studies with more targeted hypotheses.

**Comment (L273–274):** Please delete the first sentence of this paragraph.

**Response:** Thank you for this comment. We have chosen to retain the sentence as it provides transparency on the qualitative data management and analysis process, which may be especially helpful for readers less familiar with such methods.

**Comment (L335):** Please delete "only."

**Response:** Thank you. The word "only" has been removed as suggested.

**Reviewer #3: Methods Section Evaluation:**

Objectives and Hypothesis: The objectives of the study are adequately stated in the abstract and introduction. In particular, the study seeks to understand the socio-economic impacts of anthrax outbreaks and associated stressors such as food insecurity and livelihood vulnerabilities among rural Zambian communities. No formal, explicit testable hypotheses are presented, but the study successfully explores the possible relationships between anthrax outbreaks and their links to livelihood strategies as well as food security through the methods used.

Response: thank you for this commendation.

Study Design: In view of the complexity and heterogeneity of factors affecting the socio-economic burden of anthrax, a mixed-methods approach using quantitative surveys and qualitative focus group discussions simultaneously is appropriate. This design takes a mixed-methods approach that marries a broad range of data collection with in-depth qualitative insights to create rich perspectives.

Response: thank you for this comment

Population Description: The population definition is specific, being rural communities in designated Zambia GMAs. The appropriateness of this choice relies upon the frequency of anthrax and the interactions of humans, wildlife, and livestock in these designated regions, which in the introduction, are characterized as considerable.

Response: thank you for this comment

Sample Size: The paper provides a sample size calculation for the quantitative survey, using the formula for unknown populations. The rationale for the qualitative sample size (6 focus groups) is included in relation to data saturation. Although these group sizes aren't small, the lack of raw data (which might be available from the authors) and/or a power calculation means no final conclusions can be drawn.

Response: thank you for this comment. We have provided raw data

Statistical Analysis: The authors claim appropriate statistical analyses were used to screen the quantitative data by performing chi-squared tests for associations between categorical variables. Details of approaches to qualitative data analysis, such as thematic analysis based on focus groups, are provided. But more detailed information about the tests used would have provided greater transparency.

Response: thank you for this comment. Only descriptive statistics and chi square test were used due to the exploratory nature of the study.

Ethics and Permission: This study was ethically approved by ERES Converge, while anonymization processes are described in detail to demonstrate compliance with ethical standards. These ensure the anonymity of all participants and state the right to leave the study at any time.

Response: thank you for this comment

Overall Assessment: The methodology section of the research paper is strong, with a clear study design and strong ethical considerations. The sample size estimates are believable and persuasive, but a power analysis would add to the robustness. Though the statistical methods used are appropriate for the data collected, reporting the statistical tests used and further elaboration on the analysis would add rigour. The section provides a very detailed methodology and reasonable research approach.

Response: thank you for this comment

**Results:**

Reviewer #2: The results match the methods but are presented in a lengthy and suboptimal way. Figures and tables need improvement.

Response: thank you for this comment. We have improved the tables

**Detailed comments:**

As demographic characteristics are detailed in table 1 (should be table 3 if table 1 and 2 are to be kept), the text in 3.1.1. could be condensed more. The p values are not needed in table 1, please delete this column, it only serves to show that the participants were different in some aspects and not others but contributes no scientific meaning.

Response: thank you for these comments. The p-values of the chi square test have been removed.

Table 2 (or table 4?): here, and in other places in the text, it would be useful (or even necessary) to clarify what animal species are indicated in the term livestock (as this differs in different parts of the world), particularly as there is specific mentioning of poultry as an alternative in some places and as not all species are equally susceptible to anthrax. In addition, ‘charcoal burning’ requires further explanation for the uninitiated reader, how do people make money out of burning charcoal? As for the household income, the currency must be stated and it should also be related to the mean national income and the poverty threshold to improve understanding. The coping activities also need explanation, how can gardening be useful if there is a draught? And livestock farming (that is already a main or alternative livelihood for nearly half of the respondents? The last row could be named ‘support mechanism’ or similar, to differentiate form the row above that also uses the word cope. (All explanations asked for should be added to the text, not the table)

Response: thank you for the comments. This has been explained clearly in the results. We have include the species of livestock in text. The currency Zambian Kwacha (ZMW) has been added with a note on exchange rate to the USD included (1USD-ZMW28). The mean national income has been included in the discussion where this has been explained. Livestock can be main source of income to others and alternative source to others hence appearing on both. Support mechanism has been adopted

L362: does farming mean crop farming here? Please add some clarification

Response: yes it means crop farming and it has been added

L 376-377: the statement appears contradictory, if they could not sell their animals, why couldn’t they get food form them? Was it the death of the livestock that was the problem, not the failure to trade?

Response: Our previous studies have shown that the main purpose of keeping livestock is not meat but draught power, transport, milk etc. slaughter for meat is the last option is mostly when animals die due to disease and other conditions (1,2).

1.	Mumba C, Skjerve E, Rich M, Rich KM. Application of system dynamics and participatory spatial group model building in animal health: A case study of East Coast Fever interventions in Lundazi and Monze districts of Zambia. PLOS ONE [Internet]. 2017 Dec 15 [cited 2018 Oct 29];12(12):e0189878. Available from: https://journals.plos.org/plosone/article?id=10.1371/journal.pone.0189878

2.	Mumba C, Häsler B, Muma JB, Munyeme M, Sitali DC, Skjerve E, et al. Practices of traditional beef farmers in their production and marketing of cattle in Zambia. Trop Anim Health Prod. 2018;50(1):1–14.

Although I realise that the above are quotes from the FGDs, they must have been said in a context that provided more comprehension and that should be provided if the quotes are to be kept.

Response: thank you for the comment. However, the standard for qualitative study is that quotes must be presented verbatim. The interpretation is what provides more comprehension.

L 405: is ‘predominantly rural’ a result, didn’t you specifically recruit from a rural population? The results from cross-checking of demographic characteristics could be put in one or two sentences stating whether the two groups were similar or not. What is meant by ‘interplay between data sources’ (L411)? Do your results really show how socio-economic factors shape livelihoods? You have asked about climatic factors and disease

Response: thank you for the comment. All study sites were rural. We specifically recruited rural. This has been deleted.

L 438: please change from ‘will’ to ‘may’ as this consequence cannot be certain.

Response: thank you for the comment. This has been changed

3.3.4. this section should either be expanded, if there are sufficiently detailed results to do so, or removed as it is currently too weak.

Response: Thank you for the comment. The section has been deleted.

I find the Results a little scattered and would suggest re-organising into headings such as: Demographics (very brief section only to demonstrate the participant characteristics); Food security and impact of anthrax and draught, respectively, and; Livelihood diversification. Under these headings there could be subheadings for qualitative and quantitative results. I believe that would improve the flow of the text and emphasise the overall results better.

Response: thank you for the comment. We feel that this will complicate the flow of results because in the current format, we have quantitative results, then qualitative results and finally validating quantitative results with qualitative. This is the standard for conducting mixed methods study designs. Changing this will affect the flow of results.

**Reviewer #3: Variance Analysis:**

The text indicates that the analysis performed matched that of the analysis plan. Qualitative data were analyzed using thematic analysis, which is described in the Methods section below, while the quantitative data were analyzed using suitable statistical tests (chi-squared tests referred to in the text). This indicates a strong alignment of the planned and implemented analyses.

Response: thank you for this comment

**Clarity and Completeness of Results:**

Results are clearly presented, with findings primarily presented as quantitative or qualitative. Main findings are summarized, and tables referenced to describe socio-demographic data, but they are not complete tables, only parts of them. Illustrative quotations also represent the qualitative results well. The level of detail seems adequate given the constraints of a research article page.

Response: thank you for this comment

**Figures and Tables:**

Referring to Tables 1 and 2 in the text: only parts are visible. Decent tables of this type should provide sufficient quality in communicating the quantitative findings. It was unclear if the quality of the visible sections would have been maintained across a full display. As for figures, both Figure 1 and 2 are referenced in the text but not fully shown (impossible to assess quality). The graphics seem relevant based on the text, and the text needs to provide more detail to be able to display the clarity of any visual aids.

Response: thank you for this comment. The maps (figure 1 and 2) have been replaced with clear ones

**Overall:** The results section is good with a good overall presentation of the findings. The narrative demonstrates the findings are articulated clearly and sufficiently. This is a clear example of the alignment between analysis plan and execution. However, a full assessment of the quality of the figures and tables would take the full supplementary materials, which were not provided.

Response: thank you for this comment. The tables and figures have been taken to supplementary text.

**Conclusions:**

Reviewer #2: I have comments on both Discussion and Conclusion, see below. Conclusions are not supported by presented data and limitations are not described. the authors discuss their results in relation to the context and address public health relevance.

**Response:** Thank you for this observation. We have strengthened the conclusion and study and include limitations.

**Discussion**

**Comment (L459):** Are the livestock practices high-risk? How so? Please clarify.

**Response:** Thank you for this important question. We have revised the sentence to clarify how certain livestock practices, such as handling carcasses or grazing in contaminated areas, may increase the risk of anthrax.

L462-463: the statement appears totally unrelated to the cited reference, pleased check and revise or remove

**Response:** Thank you for pointing this out. We have replaced the reference with one that appropriately supports the revised statement.

L463-465: this is also not related to the reference cited, please revise or remove

**Response:** The cited reference has been removed, and the sentence revised for clarity.

L466-468: please provide some evidence/reference for the statement about older farmers.

**Response:** Thank you for the comment. The statement is not supported so it has been deleted.

L468-470: the cited reference does not cover preventive animal health, it deals with HIV. Please explain how this relates to the statement.

**Response:** Thank you for the comment. The reference has been deleted

L475-476: how does charcoal burning disturb anthrax spores? Does it involve digging? Burning would destroy the spores so the statement is confusing.

Response: we have included this paragraph, “While diversification offers financial stability, activities such as charcoal burning disturb anthrax spores in soil, increasing exposure risks (25) charcoal burning it aids in exposing the anthrax spores as it involves, cutting of trees, uprooting of roots and disturbing top soils which can expose dormant anthrax and make them more accessible to human and livestock and can alter soils condition (26). Furthermore, burning charcoal causes deforestation, which diminishes natural habitats and brings wildlife closer to areas utilized for cattle grazing and human habitation. Increased contact may make it easier for anthrax to spread, particularly if the bacteria is present in wildlife. According to a research conducted in Zambia's Chama District in the Eastern Province, where an anthrax outbreak occurred and resulted in 511 human cases and 85 hippopotamus deaths, environmental changes and food deprivation caused animal and human activity to come into closer contact (27)”.

L 479: please provide information about mean/median income and poverty threshold to improve understanding of these figures

Response: According to the Zambia Statistics Agency's living conditions monitoring survey, the average monthly household income in rural areas was estimated to be K2112.20 (less than $100), while the poverty threshold revealed that 60% of Zambians lived below the national poverty line, with a range of 78.7% in rural areas and 31.9% in urban areas (28). International poverty returns include 93% of Zambians living below the national poverty line. As of 2022, the average Zambian lives on less than $5.50 a day(29). These savings are primarily managed through mobile and village banking.

L 480: what formal mechanisms are there, if mobile banking and village banking are categorised as informal?

Response: mobile banking and village banking are both formal. We have revised it accordinglys

L484: the cited reference is about electric vehicles and does not relate to the statement. Nevertheless, it is not clear how farmers interacting with wildlife would increase their risk of anthrax. Slaughtering of animals (wild or domestic) that have died of anthrax, or digging in the ground and creating spore-containing aerosols would be potential ways of getting anthrax in this setting but that is a rather indirect ‘interaction’ with wildlife.

Response: thank you very much for a giving the paper a thorough eye. This has been deleted.
L 492: please remove name of reference. This reference focuses on anthrax in animals and should be used as a basis for writing about transmission in the setting chosen for the study. It does not, however, back up the statement preceding it on L490-491.

Response: the reference has been deleted

L 493 and L 495: please explain how gardening can be a coping strategy when there is a dry spell, wouldn’t gardening be a challenge with water shortage?

Response: we have included this paragraph, “Although gardening typically requires access to water, participants identified it as a viable coping strategy during dry spells due to the availability of residual moisture near riverbanks, floodplains, or hand-dug wells in certain locations. In these areas, communities were able to grow fast-maturing vegetables or drought-tolerant crops on a small scale for household consumption and local sale. This form of gardening served as a supplementary food and income source, particularly when rainfall-dependent crops failed. However, its feasibility varied depending on local water access and was not uniformly available across all sites”.

L499-500: you didn’t investigate climatic shocks, you asked about draught, please remove or rephrase.

Response: this has been corrected

L502: the cited reference deals with geospatial analysis and cannot be used as a basis for the preceding statement.

Response: this has been corrected

L 504-505: this statement contradicts the one that said that livestock management is high-risk, here it appears that alternative practices are more risky.

Response: Thank you for the comments. We have since revised the sentence to read as” The reliance on immediate income-generating activities such as charcoal burning, fishing, and small-scale businesses often diverts attention and resources away from vaccinating livestock against diseases, increasing the risk of anthrax transmission and maintenance”

**Conclusions**
This section does not draw clearly on the results of the study, it is more speculation than conclusion (although not necessarily wrong).

**Response:** Thank you for this observation. We have strengthened the conclusion and study and include limitations.

**Reviewer #3:** Data Is in Support: The findings seem adequately supported by the data provided. The discussion section more closely connects findings derived from both the quantitative and qualitative analyses to the conclusions drawn based on the analysis, indicating key relationships between anthrax outbreaks, livelihood diversification, food insecurity, and vulnerability among the study population.

Response: thank you for this comment

**Limitations:** The limitations of the analysis are not explicitly stated in the conclusion section provided. Although the discussion section likely contains more explicit mention of limitations (e.g., limitations in the sample size or the reliance on self-reported data), the conclusion itself should briefly mention major limitations that impact the overall interpretation of results.

Response: thank you for this comment. We have provided a subheading 4.2 for limitations of the study

**Relevance to the Current Picture:** The discussion needs to emphasize the importance of the findings, regarding food security and livelihoods, especially in the backdrop of anthrax outbreaks in GMAs. The highlighting of vulnerabilities in these groups suggests specific interventions that can be implemented in order to build greater resilience at the community level.

Response: thank you for this comment

**Public Health Relevance:** The public health relevance is well articulated, indicating the importance of interventions for controlling anthrax outbreaks, diversifying livelihood, and increasing food security. The study emphasizes the importance of the risks of zoonotic diseases in rural settings and reminds us that advice cannot simply be given but rather should be community-based, including empowerment among community members, especially women.

Response: thank you for this comment

Overall: I felt that the conclusions section was reasonably well-written, but I think the authors should more clearly and succinctly state the limitations of the study and should more directly articulate the public health implications and impact of the work. The current conclusion might go on more strongly if it described the takeaway and impact or implication of the study's findings in a more direct way.

Response: thank you for this comment. The conclusion has been revised.

**Editorial and Data Presentation Modifications?**

Reviewer #3: Given the information provided, the paper appears to be of good quality. The methodology is sound, the analysis is appropriate, and the results are clearly presented. The areas needing the most attention are relatively minor and primarily editorial in nature. Therefore, I recommend a Minor Revision.

Response: thank you for this commendation.

**Here are specific editorial suggestions:**

**Abstract:** While the abstract is good, consider adding a concise statement summarizing the main quantitative finding (e.g., "…87.9% of households were adversely affected by drought, highlighting significant economic vulnerability"). This would make the key finding more impactful early on.

Response. Thank you for this comment. This has actually been brough earlier in the abastract as a key finding.

**Results:** Fully present Tables 1 and 2. The current presentation of snippets isn't sufficient. Include all columns and data for a complete and clear understanding. Similarly, if Figures 1 and 2 are crucial for understanding, ensure they are of high quality and included in full. If they are less critical, consider whether their inclusion adds necessary value.

Response: thank you for this comment. they have been replaced with a clear figure.

**Discussion:**

Explicitly state the limitations of the study in the discussion section. This might include limitations on sample size, generalizability to other regions, the reliance on self-reported data, or any other factors that could affect the interpretation of results. Summarize these limitations concisely within the conclusion section itself.

Response: thank you very much. We have since included a section on implication of the study finding and limitations of the study.

**Conclusion:** Strengthen the concluding statement by summarizing the key implications of the findings in a more direct manner and emphasizing the overall impact of the study. For example: "This study demonstrates the critical interconnectedness of anthrax, food insecurity, and livelihood strategies in rural Zambian communities. Tailored interventions emphasizing community empowerment, livelihood diversification, and access to resources are urgently needed to mitigate zoonotic disease risks and improve community resilience."

Response: Thank you very much for this suggestion and literally rewriting out conclusion. This has been revised and included in the conclusion section.

**Overall Style:** The writing style is mostly clear. However, a careful review by a native English speaker and/or professional editor is recommended to polish the language, ensuring grammatical accuracy and conciseness. By addressing these minor revisions, the manuscript would be substantially improved and ready for acceptance.

Response: thank you for this comment. The grammar has been improved using a premium version of Grammarly software.

**Summary and General Comments:**

**Reviewer #2: Summary:**

The paper covers an interesting and important topic. The approach is good but the paper lacks clarity and needs to be rewritten.

There appears to be a lot of assumptions that are not clearly stated and evidenced in the text, many things need to be clarified for the paper to be useful for the reader.

Response: thank you for this comment. We have since clarified most statements as suggest by the reviewers. See the revised manuscript with tracked changes.

**Reviewer #3: Summary:**

This study offers an important mixed-methods exploration into the socio-economic consequences of anthrax outbreaks in rural Zambian communities residing in Game Management Areas (GMAs). It utilizes both quantitative surveys and qualitative focus group discussions to give a well-rounded view of the complex interplay of factors involved. The analysis is sound, and, although the results are not entirely represented in the text included in the R&R, they are well-presented and support the conclusions. They identify the pandemic-related vulnerabilities of these populations and assert the necessity of adaptation strategies for the response efforts. The public health implications are far-reaching.

Response: thank you for this comment

**Strengths:**
Integrating the Two: By combining quantitative and qualitative data, this now provides the richness of data and depth of understanding through breadth with the integration of the two.
Importance: The study addresses an important public health issue in a vulnerable population.
Contextual Understanding: The study is largely successful at placing the anthrax challenge among the wider issues of livelihoods, food security, and environmental constraints to success in GMAs.

Response: thank you for this comment

Ethical Approval: The study obtained ethical approval from the relevant institutional review board, and informed consent was obtained from all participants.

Response: thank you for this comment

**Weaknesses:**
Limited Explicit Discussion of Limitations: The Discussion likely describes limitations of the study, but they are not clearly enumerated in the conclusion or abstract. This should be rectified.

Response: thank you for this comment. The limitations have been clearly outlined.

Potential for Better Quality: Although generally well-written, professional editing would improve its flow and brevity.

Response: thank you for this comment. This has been done in respective sections.

**Novelty and Significance:**

In understanding the socio-economic impact of anthrax in the context of Zambian GMAs, the study contributes significantly. Although anthrax has been studied elsewhere, this research provides important local perspectives, particularly on the links between livelihoods, food security, and zoonotic disease risks. The mixed-methods approach and assessment of community-specific vulnerabilities further improve the novelty of the findings.

Response: thank you for this comment.

**General Execution and Scholarship:**

The study is a marker of a strong scholarship of high value, applied by correct methods and followed with an appropriate reading of the data. The authors demonstrate a good knowledge of the research topic. However, the incomplete presentation of results as well as the absence of limitation discussions here detract a bit from the overall impression and clarity.

Response: thank you for this comment. The table were overlapping but the have been improved and results fully explained.

**Recommendations:**
I recommend Minor Revision. The reason is the partial presentation of the main results (Tables 1 & 2, and the Figures). These must be fully incorporated to accommodate for an accurate assessment.

Response: thank you for this verdict.

**In addition to that:**

All Data Shown: Ensure all data in Table 1 and 2 and Figure 1 and 2 are clearly presented.
Discuss Limitations: Discuss the limitations of the study (e.g., sample size, generalizability, reliance on self-reported data) explicitly in both the Discussion and the Conclusion sections.
Response: thank you for this comment. The limitations have been fully discussed.

**Chisoni Mumba, PhD**

On behalf of the authors
